# The age-dependent influence of self-reported health and job characteristics on retirement

**DOI:** 10.1007/s00038-012-0411-8

**Published:** 2012-09-25

**Authors:** Dimitri Mortelmans, Jorre T. A. Vannieuwenhuyze

**Affiliations:** University of Antwerp, Antwerpen, Belgium

**Keywords:** Survival analysis, Measurement bias, Health, Retirement, ECHP

## Abstract

**Objectives:**

Social scientists and economists doubt the usefulness of self-reported health status as an indicator of overall health status. Self-reported health acts as a justification for retirement when this decision is in reality driven by other reasons. In this study, we looked at income, job satisfaction, and job status.

**Methods:**

We introduce a survival model (Cox model) that simultaneously includes both health and job characteristics as independent variables. We also take the age-dependent character of these effects into account.

**Results:**

An analysis of the European Community Household Panel data did not validate the justification bias with respect to these variables. The addition of job characteristics had no influence on the effect estimates of self-reported health.

**Conclusions:**

We found significant effects for self-reported health as well as for objective health measures. The addition of job characteristics did not contribute to the explanation of the effect of self-reported health falsifying the justification bias hypothesis.

## Introduction

Health status has been cited as one of the major reasons for early retirement (McGarry 2004; Dwyer and Mitchell 1999; Bound et al. 1999) and it is sometimes considered even more significant than financial status (McGarry 2004). It is generally assumed that those in better health tend to retire later (Siddiqui 1997; Kalwij and Vermeulen 2008). Several factors may explain why poor health induces people to retire earlier: first, it hinders job performance, making work less rewarding. It also requires people to use their leisure time to care for their health. Finally, it also increases non-wage income, from health insurance and disability benefits, for example.

Assessing the effects of health remains particularly difficult for two reasons. First, health status is not static but dynamic, meaning that it changes over time. It is changes in health, rather than in a person’s overall health status, that have an effect on the retirement decision. This dynamism has often been overlooked in previous research (Bardage et al. 2005).

Second, obtaining accurate measures for health is problematic. Self-reported health status is often used as a measure of real health status, but this indicator may overstate the real effect of health on retirement (McGarry 2004; Dwyer and Mitchell 1999). Self-reported health tends to justify retirement while other job-related aspects, such as job satisfaction and income, determine the decision. We therefore expect the effect of self-reported health to diminish when job characteristics and objective health measures are controlled for.

### Health and retirement: a justification bias?

Defining retirement is complex. Topa et al. (2009) define retirement as both a process of preparation for retirement, or as a progressive transition, and as an abrupt switch, i.e. the decision to retire itself. However, Wang and Shultz (2010) distinguish four theoretical concepts in the retirement literature. To begin with, retirement as decision making defines retirement as a motivated choice behaviour. A well-known related theory is rational choice theory, which characterises an individual as a rational actor comparing the advantages and disadvantages of retiring. Retirement is thus the result of comparing the financial resources accumulated with those needed for retirement (Hatcher 2003; Brougham and Walsh 2005; Szinovacz and Deviney 2000). Second, retirement can be described as an adjustment process, which emphasises that it is more than a simple decision (Wang and Shultz 2010). Retirement transitions follow different pathways and are the result of a variety of contextual influences (e.g. pension programmes, security systems) and individual characteristics (e.g. health status, work ethic and job satisfaction) (Szinovacz 2003; van Oorschot and Jensen 2009). Third, retirement is defined as a career development stage. In this definition, retirement is no longer dictated primarily by the organisation, but increasingly by workers’ personal values and goals. This is also known as the protean career (Wang and Shultz 2010; Hall 2004). Finally, retirement can be considered a part of human resource management, which emphasises the influence organisations have on older workers’ retirement decisions, and which varies at organisation level according to the systematic retirement practices being employed (Wang and Shultz 2010).

Understanding the individual effect of health status on the retirement transition proves somewhat difficult (Dwyer and Mitchell 1999; McGarry 2004), and extensive academic debate surrounds the accuracy of health measures (not only in the retirement literature) (Jylha 2009). Health status is commonly measured by respondents’ subjective self-assessment, the validity of which has been questioned because respondents’ health reports may themselves be influenced by labour market situations. Several studies have examined the interplay between labour market status and health (e.g. Bartley et al. 2004; Monden 2005). Labour market status appears to have a significant effect, in that poor health is clearly associated with non-working status. Ki et al. (2011) also demonstrate that health associations among working people are far less significant than among non-working people.

While poor health may rationalise the retirement decision, self-reported health indicators may also exaggerate the effect of health on retirement, since leisure preferences also play a substantial role in the decision (Kerkhofs et al. 1999; Dwyer and Mitchell 1999; Kalwij and Vermeulen 2008; McGarry 2004). This is called the justification bias (McGarry 2004; Dwyer and Mitchell 1999). A number of studies have indeed found that self-reported health exaggerates the impact of health on the retirement decision: some respondents actually reported a worse self-assessed health after retirement than before (Anderson and Burkhauser 1985). Other studies have found no empirical support for the justification bias whatsoever (Dwyer and Mitchell 1999; Kalwij and Vermeulen 2008). The study of Au et al. (2005) even produced an underestimated effect of health when using self-assessed health status as a measure, which indicates a bias in attenuation rather than in justification.

The justification bias implies that the effect of self-reported health is influenced by other factors, such as working status. Subjective health measurements are also influenced by real health status, however, which does make the measure useful as an indicator of health. Previous research has revealed the distinct effects of subjective (Kalwij and Vermeulen 2008; McGarry 2004; Miah and Wilcox-Gok 2007) and objective measures (Disney et al. 2006; Heyma 2004; Kalwij and Vermeulen 2008; McGarry 2004). However, objective measures of health cannot fully explain the relation between subjective health indicators and retirement, and the effects of the latter measures—when the former are controlled for—may indicate a justification bias.

In summary, these theoretical considerations suggest that any association between self-reported health and retirement is illusory. Both are influenced by real health status and by other job-related determinants. We therefore expect that during simultaneous analysis of the effects that job characteristics and health have on retirement, the effect of subjective health measures will diminish when objective measures and job characteristics are controlled for.

### Modelling retirement as a dynamic process

To date, retirement studies that include both health and job indicators have not provided entirely satisfactory results. In fact, it is not poor health but deteriorating health that tends to push individuals towards retirement (Bound et al. 1999). Models that employ age and activity status as dependent variables have commonly been used to analyse the relationship between health and the likelihood of being retired or inactive (e.g. Au et al. 2005; Kalwij and Vermeulen 2008; Shultz and Wang 2007), which has resulted in an underrepresentation of job characteristics. Moreover, questions may be raised over the direction of causality in such analyses, since a significant effect of health on retirement demonstrates only a relation between the two, and no causal direction (Lindeboom and Kerkhofs 2009).

Retirement is an event that usually occurs only once in an individual’s lifetime. The statistical framework needed to model the occurrence of such an event is a survival or hazard model, and research that has been conducted on the relationship between health and retirement using this hazard model is scarce. Disney et al. (2006) employed a hazard model to investigate the relationship between health shocks and retirement in the UK, but job characteristics were excluded from the model. Zissiniopoulos and Karoly (2007) and Kerkhofs et al. (1999) employed hazard model that included both subjective health variables and job characteristics, but Zissiniopoulos and Karoly made no clear distinction between objective and subjective health measurements. Kerkhofs, Lindeboom, and Theeuwes found that subjective measures did indeed overestimate the effect of health in comparison to objective measures. However, these studies all make the assumption that the effects of health and other variables remain constant across age groups.

## Methods

### Data

The analyses were performed using the European Community Household Panel (ECHP). This international micro-database includes data for 15 Western European countries and was set up by Eurostat. An important feature of the ECHP is its longitudinal character: data are available for eight annual waves between 1994 and 2001. This feature enables us to track changes in health status over time and to model exact retirement dates.

Respondents between the ages of 50 and 65 were selected. Only those active on the labour market at the age of 50 were considered, since inactive people are not part of the risk set. We set 65 as the upper limit for age in our analysis since this was the maximum official pension age for all countries included in the database. The ECHP analyses a random sample of individuals, but all other individuals in the household are also surveyed. Our sample was restricted to the reference person in order to avoid dependencies. Respondents from Germany and Luxembourg were excluded because too many of the variables we wished to investigate were lacking. Respondents from the Netherlands were also excluded because only 21 respondents retired during the period of study. Sweden had no panel data in the ECHP. The total number of respondents in the final sample was 13,434 and 11 countries were represented: Austria, Belgium, Denmark, Spain, Greece, France, Finland (from 1996 onwards), Italy, Ireland, Portugal and the UK. Finally, we excluded data from 1994 because too many variables of interest were not surveyed in that wave. In total, 41,810 data lines (person–wave combinations) were available for analysis. Table 1 provides an overview of the respondents available in each country, by wave.

**Table 1:** Distribution of respondents by wave and country, number of events by country

Country	Wave							Total	No. of events
	1995	1996	1997	1998	1999	2000	2001		
Austria	545	737	699	661	627	596	498	4,363	401
Belgium	463	552	547	560	548	511	427	3,608	198
Denmark	643	748	693	665	660	676	607	4,692	223
Spain	1,170	1,398	1,257	1,236	1,126	1,110	939	8,236	329
Greece	1,148	1,359	1,302	1,192	1,046	1,005	909	7,961	375
France	1,111	1,389	1,314	1,257	1,261	1,258	1,093	8,683	556
Finland		1,159	1,408	1,348	1,282	1,052	949	7,198	372
Italy	1,397	1,742	1,649	1,599	1,533	1,451	1,220	10,591	688
Ireland	695	799	754	708	624	553	467	4,600	139
Portugal	1,419	1,568	1,499	1,475	1,439	1,410	1,230	10,040	395
UK	836	1,009	1,090	1,104	1,109	1,107	987	7,242	306

European Community Household Panel (1995–2001)

### Variables

The ECHP includes a question about the respondent’s main activity status, where one of the response categories is ‘retired’. This response was used to obtain information about retirement age.

In order to measure subjective health, we used a five-point Likert scale to represent the respondent’s opinion on his/her own health in general (from ‘very good’ to ‘very bad’). Next, ECHP asked whether the respondent had been admitted to a hospital as an in-patient during the last year, how many nights they had spent in hospital during the last year, and the number of times they had consulted a GP or medical specialist during the last year. These variables were treated as objective health measures and were scored so that higher scores corresponded to poorer health.

Jobs were characterised using two variables. One represented the respondent’s job status, and was divided into six categories: (1) ‘legislators and managers’, (2) ‘professionals’, (3) ‘white-collar workers’, (4) ‘skilled blue-collar workers’, (5) ‘unskilled blue-collar workers’, and (6) ‘unemployed’. Job satisfaction was also added to the analysis and was measured on a six-point scale from ‘not satisfied’ to ‘completely satisfied’.

Next, we considered the respondent’s income. Since income is largely determined by occupation, it could also be considered a job characteristic. However, it is more likely to be access to capital than income itself which affects the decision. We therefore included net disposable household income rather than personal income (both types of income were surveyed). Distribution of this variable fluctuates significantly between countries. Since it is differences between respondents within one country (and not between countries) that will have an influence on retirement, we standardised this variable within each country.

Finally, we added a number of control variables: gender, cohabitation status (living as a couple or not) and educational level. Educational level consisted of three categories: ‘tertiary’, ‘secondary’, and ‘did not complete secondary school’.

### Imputation

As is often the case with longitudinal data, the ECHP had some missing values. Table 2 summarises the frequencies of missing data for all variables. At least one variable was missing in more than 23.4 % of samples, but half of these were missing by design.

**Table 2:** Frequency of missing data

	Missing by design (%)	Non-response (%)	Total missing (%)
Education level	0.4	0.5	1.0
Household income	–	3.9	3.9
Job status	1.3	3.3	4.6
# Nights in hospital	–	0.4	0.4
# Visits GP	9.8	0.4	10.2
# Visits med. specialist	10.9	0.5	11.4
In-patient in hospital	–	0.3	0.3
Cohabitation	0.1	0.1	0.1
Job satisfaction	0.5	3.9	4.4
Subjective health	0.5	0.3	0.8
Total missing	11.7	11.7	23.4

European Community Household Panel (1995–2001)

To compensate for the missing information, we performed multiple imputation (MI) [see also Rubin (2004), Schafer (1997)]. Here, *Y* represented the complete data set, *Y*_obs_ the observed values of, *Y*, and *Y*_mis_ the missing values. *θ* represented the parameter vector of the data model for *Y*. MI consists of three steps:

1. First, the missing values are imputed $m \ge 2$ times, using an appropriate model dependent on that incorporates random variation: $P\left( {Y_{\text{mis}} |Y_{\text{obs}} } \right)$. This results in complete data sets. Estimation of missing values is performed using a Gibbs-sampler, which is a Markov Chain Monte Carlo (MCMC) technique.
2. The desired analysis is performed on each complete data set using a complete-data technique. In our case, the complete-data model was a cloglog-model, which is explained in the next section. The parameters were estimated using $P\left( {\theta |Y_{\text{mis}} ,Y_{\text{obs}} } \right)$.
3. The average of the parameter vectors of interest is calculated. The parameters’ standard error can be obtained using the average squared standard error of the parameter estimates and the variance of the parameters. These parameters are reported in Table 3.

**Table 3:** Parameter estimates, standard errors, and test information

	Estimate (SE)		
	Model 1	Model 2	Model 3
Intercept			
AU	−3.903 (0.163)***	−4.194 (0.170)***	−5.348 (0.295)***
BE	−4.148 (0.193)***	−4.357 (0.196)***	−5.534 (0.308)***
DA	−5.323 (0.251)***	−5.434 (0.251)***	−6.591 (0.351)***
SP	−7.588 (0.291)***	−7.714 (0.292)***	−8.845 (0.367)***
GR	−4.489 (0.155)***	−4.598 (0.156)***	−5.500 (0.249)***
FR	−4.425 (0.134)***	−4.549 (0.135)***	−5.644 (0.261)***
FI	−5.080 (0.230)***	−5.196 (0.231)***	−6.384 (0.329)***
IT	−3.657 (0.112)***	−3.837 (0.117)***	−4.880 (0.244)***
EI	−4.838 (0.245)***	−4.963 (0.246)***	−6.042 (0.336) ***
PO	−4.554 (0.167)***	−4.613 (0.167)***	−5.597 (0.267) ***
UK	−5.017 (0.201)***	−5.121 (0.201)***	−6.135 (0.301) ***
Age-50			
AU	0.308 (0.019)***	0.324 (0.019)***	0.406 (0.031)***
BE	0.245 (0.019)***	0.256 (0.020)***	0.324 (0.031)***
DA	0.335 (0.022)***	0.340 (0.022)***	0.412 (0.033)***
SP	0.497 (0.023)***	0.502 (0.023)***	0.580 (0.032)***
GR	0.231 (0.013)***	0.235 (0.014)***	0.304 (0.023)***
FR	0.326 (0.013)***	0.331 (0.013)***	0.402 (0.026)***
FI	0.316 (0.022)***	0.323 (0.022)***	0.400 (0.032)***
IT	0.171 (0.011)***	0.180 (0.011)***	0.253 (0.024) ***
EI	0.216 (0.022)***	0.222 (0.022)***	0.297 (0.032)***
PO	0.189 (0.015)***	0.190 (0.015)***	0.262 (0.025)***
UK	0.281 (0.019)***	0.285 (0.019)***	0.353 (0.029)***
Subjective health^a^	1.089 (0.144)***	0.690 (0.160)***	0.682 (0.164)***
Age-50	−0.056 (0.014)***	−0.032 (0.016)*	−0.036 (0.016)*
# Consulted GP		0.027 (0.007)***	0.027 (0.007)***
Age-50		−0.001 (0.001)	−0.002 (0.001)*
# Consulted specialist		0.023 (0.009)**	0.024 (0.009)**
Age-50		0.000 (0.001)	0.000 (0.001)
In-patient		0.430 (0.165)**	0.409 (0.168)*
Age-50		−0.036 (0.018)*	−0.034 (0.018)
# Nights in hospital		−0.003 (0.006)	−0.002 (0.007)
Age-50		0.001 (0.001)	0.001 (0.001)
Job satisfaction			0.131 (0.042)**
Age-50			−0.007 (0.004)
Household income			0.096 (0.046)*
Age-50			−0.015 (0.005)**
Manager			−0.656 (0.225)**
Age-50			0.035 (0.020)
Professional			0.138 (0.161)
Age-50			−0.001 (0.016)
White collar			0.346 (0.150)*
Age-50			−0.018 (0.015)
Skilled blue collar			–
Age-50			–
Blue collar			0.271 (0.154)
Age-50			−0.016 (0.015)
Unemployed			0.873 (0.185)***
Age-50			0.011 (0.018)
Male	0.072 (0.057)	0.092 (0.057)	0.100 (0.058)
Partner	−0.030 (0.055)	−0.034 (0.055)	0.031 (0.055)
Third level education	−0.249 (0.058)***	−0.243 (0.058)***	−0.157 (0.074)*
Second stage education	0.087 (0.051)	0.084 (0.051)	0.101 (0.054)
<Second stage education	–	–	–

European Community Household Panel (1995–2001)

^a^Dichotomized

* *p* < 0.05

** *p* < 0.01

*** *p* < 0.001

Imputations were performed using the PAN software written by Schafer (2002), which can be downloaded as an R package. Given that several variables were not measured in all countries (e.g. objective health), no country effects could be added to the imputation model since this would result in unobserved associations and ambiguity in the distribution of missing data. As a consequence, we implicitly assumed that the relation between the other variables was comparable across countries, and associations from one country were then used to fill in missing values for other countries.

### Model

Our analysis employed a Cox model to model the hazard, *h*_*it*_, of individual at age *t*,

$$ h_{it} = (T = t|T \ge t,\eta_{it} ,j_{it} ,x_{it} ),\;t = 50,51, \ldots ,65,\;\,i = 1, \ldots ,N. $$

$\eta_{it}$ represents the real health status of individual *i* at age *t*, *j*_*it*_ are his/her job characteristics (satisfaction, status) and stands for other personal characteristics. The model is as follows:

1 $$ {\text{cloglog}}\left[ {h_{it} } \right] = \beta_{ot} + \eta_{it} \beta_{1t} + j_{it} \beta_{2t} + x_{it} \beta_{3t} + \varepsilon_{it} $$

It has the following complementary log–log link function:

$$ {\text{cloglog}}\left[ {h_{it} } \right] = { \log }\left( { - { \log }\left( {1 - h_{it} } \right)} \right) $$

Since $\eta_{it}$ cannot be observed, it has been replaced by certain indicators. *s*_*it*_ stands for the subjective health indicator, and this indicator captures with error.

2 $$ s_{it} = \eta_{it} + v_{j,it} + v_{1it} $$

We factor the error into two terms: $v_{j,it}$ and $v_{1it}$. The first of these represents the measuring error due to the justification bias. If higher scores on $\eta_{it}$ and *s*_*it*_ represent poorer health, we expect $v_{j, it}$ to be positive on average. Indeed, the theory suggests that self-reported health exaggerates real health status because of other job-related retirement incentives. *v*_1*it*_ represents the remaining measurement error due to inaccurate measurement. We assume *v*_1*it*_ to be normally distributed with mean zero and independent of *T* or $h_{it}$. $s_{it}$ can also be written as follows:

3 $$ s_{it} = \lambda_{1} o_{it} + \lambda_{2} j_{it} + v_{2it} $$

$o_{it}$ represents objective health indicators; *v*_2*it*_ represents variability in *s*_*it*_ which cannot be explained by objective health measures or job characteristics. Regarding the justification bias, we expect *v*_*jit*_ and $\lambda j_{it}$ to be equal. Then, $v_{2it}$ reduces to the error of the objective health measurements in measuring $\eta_{it}$. If we implement (2) and (3) in (1), we obtain

4 $$\begin{aligned}{{\text{cloglog}} [h_{it}] = \beta_{0t} + \beta_{1t} \lambda_{1}o_{it} + \beta_{1t} \lambda_{2} j_{it} + \beta_{2t} j_{it}}\\+ \beta_{1t} \left(v_{2it} - v_{j,it} - v_{1it} \right) + \beta_{3t}x_{it} + \varepsilon_{it}\end{aligned}$$

**Fig. 1:**
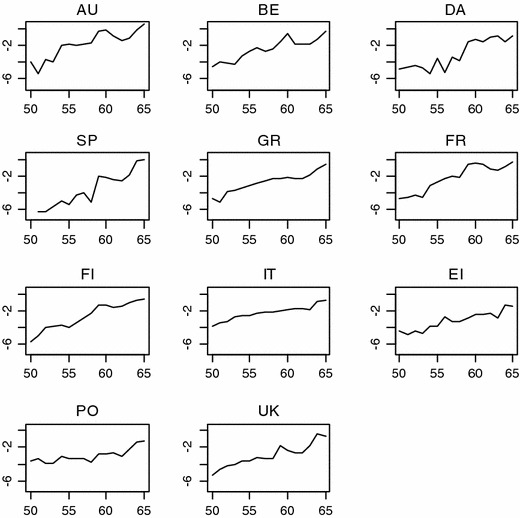
Exact cloglog hazard in the sample, by country (European Community Household Panel, 1995–2001). The cloglog function is a discrete-time variant of the Cox model, assuming proportional hazards, i.e. the hazard for one respondent is proportional to the hazard of another respondent

In this equation, $v_{2it} - v_{j,it} - v_{lit}$ represents the remaining effect of the subjective health indicator after controlling for job characteristics and objective health. Figure 1 shows the observed evolution of the cloglog hazard for each country. It is of note that this evolution is almost linear in most countries. Rather than estimating the exact hazard at each age, we reduced the model by replacing $\beta_{0t}$ in (4) by $f\left( t \right) = \beta_{0} + \beta_{a} \times t.$ The estimated baseline hazard function is then smoother (Efron 1988; Fahrmeir and Wagenpfeil 1996).

**Fig. 2:**
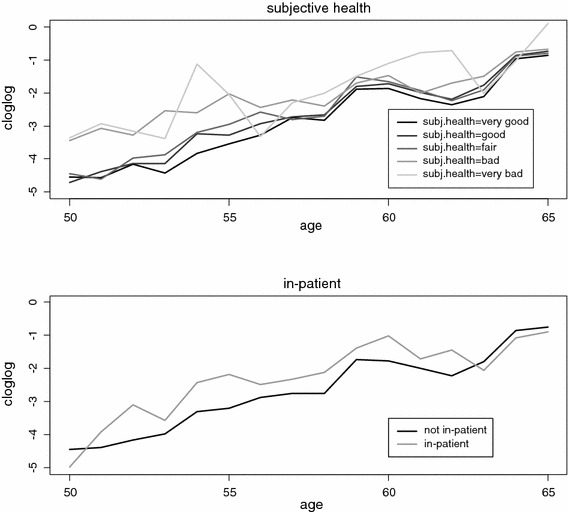
Exact cloglog hazard evolution in the sample, by health status (European Community Household Panel, 1995–2001)

Figure 2 plots the cloglog hazard for the various categories of the subjective health variable and for one objective health indicator, and demonstrates that the hazard was lower in both cases for healthy respondents than for those with poor health. A clear difference exists between hazards for younger age groups. As age increases, the difference between hazards diminishes (except for the highest age group). This particular trend was added to the analysis by including the interaction effects with age in model (4) for all health variables and job indicators.

### Country-specific (regime) effects

An important issue for the hazard models is the existence of country-specific pathways to retirement and the effects of the respective health care regimes. Besides a country-specific intercept and the age effect, we could not include country-specific effects (interaction effects) in the survival model. All assumptions made in the imputation model should be included in the analysis model (Schafer 1997). When the imputer assumes more than the analyst, biased estimates may result. No country-specific effects could therefore be added to the model, except for age (there were no missing values for country, age, or retirement status so these were not imputed).

## Results

**Fig. 3:**
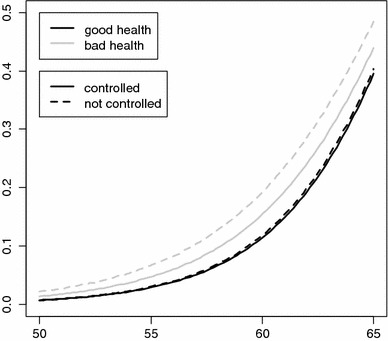
Fitted hazard curves of subjective health categories (European Community Household Panel, 1995–2001)

Table 3 presents the estimation results, which consist of three models. The first model included only the subjective health indicator and control variables. As reflected by the dashed lines in Fig. 3, individuals who felt unhealthy had a higher hazard than healthy people. Because of the negative interaction effect with age, the proportionality of the hazard decreases. If no interaction effect were present, this proportionality would be constant, resulting in an inflated hazard curve for unhealthy people.

In the second model, we added the four objective health indicators. Again, individuals in poor health have a higher retirement hazard. In contrast to the subjective indicator, these effects do not seem to decrease over the age range. Almost all interaction effects with age are very small and statistically insignificant. The effect of the subjective indicator is more interesting. In comparison with Model 1, this effect decreases by approximately one-third. The same conclusion applies to the interaction effect, which indicates that a substantial part of the correlation between self-reported health and the retirement decision may be due to real health effects.

The most interesting part of the analysis can be seen in Model 3, in which job characteristics were included. Surprisingly, higher job satisfaction correlates with higher retirement hazard and this effect remains consistent even as age increases. The same applies for income at younger ages. Higher household income is accompanied by higher hazard. However, this effect is reversed after approximately 7 years. With respect to job status, we note that managers have lower hazards and the unemployed have higher hazards than those in other categories. No appreciable interaction with age can be observed.

No difference is observed between the subjective health effects in Models 2 and 3. It appears that the relation between subjective health and retirement cannot be explained by job characteristics, including job satisfaction, which refutes the justification bias hypothesis. The relation between poorer self-reported health and increasing retirement hazard can therefore not be explained by job characteristics that push an individual toward retirement.

**Fig. 4:**
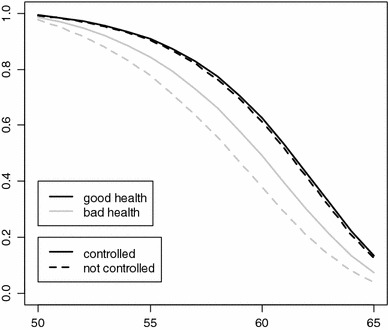
Fitted survival curves of subjective health categories (European Community Household Panel, 1995–2001)

In summary, we see a shift in the effect of self-reported health on the retirement decision when other variables are controlled for, which is mostly due to mutual dependence on individuals’ real health status. Controlling for this status reduces the effect of self-reported health. Job characteristics, on the other hand, contribute little to this shift. The nature of the shift is graphically represented by the survival functions in Fig. 4. For those who evaluate their own health as good, there is little change when objective health status is controlled for. However, for those who report poor health, the survival curve rises.

## Discussion

This article investigated the relationship between self-reported health measures, objective health measures, job characteristics and the retirement event using data from the last seven waves of the ECHP, a longitudinal cross-national survey conducted between 1994 and 2001.

The main objective of this study was to develop a model for studying the relationship between health and retirement. Since retirement is a dynamic process, a longitudinal analysis was appropriate. To date, job characteristics have largely been excluded from studies investigating the relationship between health and retirement, since they have often employed multinomial models in which activity status is considered a dependent variable. This made the inclusion of job characteristics technically impossible. We proposed a survival model that would overcome this shortcoming by assessing how the likelihood or hazard of retirement—rather than retirement status itself—is affected by certain variables.

Our data suggest that age-dependent effects exist for several retirement determinants. As an individual approaches official retirement age, other retirement determinants have increasingly less influence on the retirement decision. The ECHP data reveal that this applies for both health and for income, a fact which has often been ignored in previous studies. Our analyses show that the inclusion of interaction effects with age is crucial for presenting a clear picture of the effect of these determinants.

With respect to the relation between health and retirement, we find significant effects for self-reported health as well as for objective health measures. The relationship between self-reported health can partly be explained by real health status. Furthermore, the addition of job characteristics does little to explain the effect of self-reported health, which is inconsistent with the justification bias hypothesis. Further research should be conducted in order to reveal the nature of the remaining correlation between subjective health and retirement, controlling for objective measurements.

A number of limitations should be acknowledged. First, questions may be raised about the true objectivity of the objective health measures used in the analysis, since these were self-reported and therefore at risk of mismeasurement (Baker et al. 2003). True health is difficult to observe, and analyses are often restricted to noisy measures, as in this case. However, the weak correlations found between the objective and subjective health measures (see Table 4) may indicate that the objective indicators fail to measure precisely the same concept as the subjective indicator (i.e. a respondent’s subjective reaction to the presence of a disease may lead to different levels of subjective health appraisal).

**Table 4:** Correlations between health variables

	# Nights in hospital	# Consulted GP	# Consulted specialist	In-patient
Subjective health^a^	0.155	0.300	0.218	0.159
# Nights in hospital		0.201	0.230	0.533
# Consulted GP			0.321	0.222
# Consulted specialist				0.246

European Community Household Panel (1995–2001)* p* < 0.001 for all correlations

^a^Dichotomized

Even if the objective health indicators are good measures of real health status, their usefulness for retirement research is not guaranteed. Objective measures fail to identify the precise impact on the retirement decision because they measure health rather than work capacity (Bound 1991). When investigating the retirement decision, health should be defined as the physical or mental ability to work. Shultz and Wang (2007) researched more specific health conditions, and found a relation between major health conditions (cancer, lung disease) and retirement. Minor health conditions (diabetes, arthritis) were found to result in possible job changes. Rice et al. (2011) reported similar results for symptoms of depression and impaired physical mobility (lower limb pain and shortness of breath).

Second, attention should be paid to the definition of retirement. This study employed an indicator based on self-reported activity status to determine whether an individual was retired or not. Other definitions of the retirement event could also have been used, however, such as the collection of a pension, a decline in working-hours, etc. Deteriorating health may influence an individual’s labour market behaviour in other ways (Bound et al. 1999) and does not always result in retirement; it may also lead to job changes and to application for disability benefits.

Third, our measure of objective health was also not a precise indicator of actual health, since we mainly employed healthcare use as a measure of the objective health status of our respondents. The socioeconomic gradient in healthcare use has been documented frequently in different international settings (Davis et al. 1981; Hertzman et al. 1994). The potential bias in access to health services could not be covered completely in our model, though we included a wide variety of objective health indicators in order to obtain a closer estimate of actual health status. The indicators are not entirely independent of the socioeconomic gradient but we attempted to limit its influence as far as possible by controlling for income and by a variety of measures.

Finally, we should expect differences to occur not only among individual retirement decisions but also among different countries. This study pooled longitudinal data from the ECHP in order to examine the overall effects of age, working conditions and health measures. We could not include country-specific measures of healthcare systems or retirement arrangements, which were a problem we attempted to address using a simple control for country which employed country dummy variables. While this compensated at least partially for some country differences, it remains an important limitation of this study. Other, mostly cross-sectional studies have shown regime effects on health (Dragano et al. 2011), or the effects of both healthcare and pension systems on the retirement decision (Engelhardt 2011). Future studies could take into account the longitudinal structure of the retirement process while controlling for country effects in more depth. The major requirement for this, of course, is the availability of panel data which needs a minimum of imputation.
